# Electrochemical Study of Carbon Nanotubes/Nanohybrids for Determination of Metal Species Cu^2+^ and Pb^2+^ in Water Samples

**DOI:** 10.1155/2016/9802738

**Published:** 2016-11-01

**Authors:** Andréa Claudia Oliveira Silva, Luis Carlos Ferreira de Oliveira, Angladis Vieira Delfino, Mario Roberto Meneghetti, Fabiane Caxico de Abreu

**Affiliations:** Instituto de Química e Biotecnologia, Universidade Federal de Alagoas (UFAL), 57072-970 Maceió, AL, Brazil

## Abstract

The use of nanomaterials, such as nanoparticles and nanotubes, for electrochemical detection of metal species has been investigated as a way of modifying electrodes by electrochemical stripping analysis. The present study develops a new methodology based on a comparative study of nanoparticles and nanotubes with differential pulse anodic stripping voltammetry (DPASV) and examines the simultaneous determination of copper and lead. The glassy carbon electrode modified by gold nanoparticles demonstrated increased sensitivity and decreased detection limits, among other improvements in analytical performance data. Under optimized conditions (deposition potential −0.8 V versus Ag/AgCl; deposition time, 300 s; resting time, 10 s; pulse amplitude, 50 mV; and voltage step height, 4 mV), the detection limits were 0.2279 and 0.3321 ppb, respectively, for determination of Pb^2+^ and Cu^2+^. The effects of cations and anions on the simultaneous determination of metal ions do not exhibit significant interference, thereby demonstrating the selectivity of the electrode for simultaneous determination of Pb^2+^ and Cu^2+^. The same method was also used to determine Cu^2+^ in water samples.

## 1. Introduction

Metal pollution has become a serious threat to living organisms. Cobalt, copper, iron, manganese, molybdenum, and zinc are examples of metal species that play an important role in living organisms. They are essential in small quantities for the operating metabolism but may be harmful at excessive levels. Even small doses of very toxic metals such as arsenic, cadmium, lead, and mercury can cause serious problems for the environment and human health. The accumulation of such metals in the human body can cause diseases and disorders of the nervous, immune, reproductive, and gastrointestinal systems [1–4].

Lead is the most common metals species. The maximum legal concentration for lead in drinking water is 10 *μ*g L^−1^. It is a general metabolic poison and enzyme inhibitor. It can cause mental retardation and brain damage in young children. Lead has the ability to replace calcium in bones to form sites for long-term replacement. Reduced academic performance and behavior problems such as aggression may be associated with blood lead levels [5–7].

Copper is one of the biologically essential ions that are only necessary at trace-level concentrations, for the purpose, for example, of catalytic action in heme synthesis. However, excessive amounts will be toxic. Concentrations of copper higher than 1.0 mg L^−1^ in water causes environmental problems and concern has increased regarding long-term exposure and potentially toxic effects on human health, especially fast growing infants and young children [3, 6, 7].

The determination of copper and lead in the environment is increasingly important because of their toxic effects on plants, animals, and human beings. As metal species are not biodegradable and tend to accumulate in living tissues, their presence in drinking water or industrial effluents is a public health problem, as a consequence of the consumption of marine products and the presence of these metal ions in the aquatic food chain. More rigorous requirements for the removal of metal species from wastewater before release into the environment are therefore required, since the main sources of metal species are often anthropic activities, such as industrial processes, agriculture, and mining. Such activities may cause the release of metals into aquatic and terrestrial systems [1, 2, 4, 8, 9].

Various technologies have been employed for the determination of metal ions in different chemical systems, including living systems and the whole environment. Typical analysis of these metal ions has been based on standard spectroscopic techniques, such as inductively coupled plasma mass spectrometry (ICP-MS), atomic absorption spectrometry (AAS), and X-ray fluorescence (XRF) spectrometry. However, these techniques are time-consuming, high-maintenance, and expensive and require sophisticated instruments. Simple and rapid determination and monitoring of these metals in various types of samples are therefore of vital importance. The development of selective and sensitive methods for detection of the early pollution of trace metals ions is thus of great interest [1–4].

Electrochemical methods are simple and low-cost compared to optical techniques and can be applied in the field. They also have high sensitivity, selectivity, and simultaneous determination. Electroanalytical stripping techniques, such as anodic stripping voltammetry (ASV), cathodic stripping voltammetry (CSV), and stripping potentiometry, are very powerful methods for the determination of trace metal concentrations [1, 3–5, 9, 10].

Anodic stripping voltammetry (ASV) has demonstrated advantages such as speed of analysis and good performance with saline matrices like sea water; it is inexpensive, has good selectivity and sensitivity, and can conduct simultaneous analysis of mixtures. This is because, in stripping analysis, a preconcentration step is combined with a stripping step, thereby enhancing sensitivity and selectivity. During the preconcentration step, the metal of interest is collected onto a working electrode and, during the stripping step, the collected metal is stripped out into the solution [1, 3, 5, 9–11].

The kind of material under detection determines what a good and reliable electrochemical sensor is. Nanomaterials are attractive because of chemical and physical properties, such as size, composition, conductivity, mechanical strength, magnetism, and light absorbing and emitting properties. They have therefore brought many advantages for the development of electrochemical studies, such as signal enhancement for sensing technologies [1, 11, 12].

The field of chemically modified electrodes (CMEs) has received considerable attention, because these can exercise more direct control over the chemical nature of an electrode and are able to accumulate metal ions on the basis of the interaction of these ions with a functional group on the electrode surface. They also provide a renewable and modified surface, are inexpensive, and generate very low background current interferences. The key to obtaining a high-performance modified electrode and detection sensor is the way the nanomaterials and the sensitive film on sensors are modified and immobilized [3, 11, 13].

The electronic, chemical, and mechanical properties of nanomaterials, such as carbon nanotubes and metal nanoparticles, make them extremely attractive for electrochemical sensors in comparison to conventional materials. The extremely high surface-to-volume ratios associated with these nanostructures make the electrical properties extremely sensitive to species adsorbed on surfaces, leading to an increased mass-transport rate and fast electron transfer, providing excellent sensitivity and selectivity [1, 11, 12, 14].

Since their discovery in 1991, carbon nanotubes (CNTs), a fascinating new member of the carbon family, have attracted considerable attention, especially for use as an electrode component, owing to their excellent properties, such as increased electrode surface area, fast electron transfer rate, significant mechanical strength, and good chemical stability. These tubes consist of rolled graphene sheets, which can be single-walled carbon nanotubes (SWCNTs) or multiwalled carbon nanotubes (MWCNTs). Although carbon nanotubes exhibit great potential for the adsorption of heavy metal ions from aqueous solutions, removal efficiency, selectivity, and sensitivity remain limited [2, 4, 15, 16]. On the other hand, CNTs associated with other nanoparticles improve some properties, enhancing the mechanical properties of the electrode. Silica nanoparticle (SiO_2_NPs) based materials are robust inorganic solids with an extremely high surface area and tunable size and have found extensive applications in chemical mechanical polishing. Geometric and electronic structures of gold nanoparticles (AuNPs) supported by different substrates have been widely studied because of their high catalytic activity, surface-to-volume ratio, conductivity, and excellent biocompatibility [3, 17–20].

In recent years, use of nanomaterials for the detection of heavy metals by ASV has been attracting much attention, owing to their high adsorption capacity and sensitivity and the application of electrodes for simultaneous determination of Cu(II) and other metals ions such as Cd(II) and Pb(II) has been investigated. According to Afkhami et al., 2014, a sensor designed by incorporation of multiwalled carbon nanotubes (MWCNTs) and a new synthesized Schiff base into the carbon paste ionic liquid electrode (CPEIL) had a detection limit of 0.08 *μ*g L^−1^ [21]. A new composite electrode has been created using graphene, 1-n-octylpyridinum hexafluorophosphate (OPFP), and [2,4-Cl_2_C_6_H_3_C(O)CHPPh_3_] (L), as a new synthetic phosphorus ylide. The analytical performance of the proposed electrode has been examined using square wave voltammetry and low detection limits of 0.07 *μ*g L^−1^ for Tl^+^, 0.09 *μ*g L^−1^ for Pb^2+^, and 0.08 *μ*g L^−1^ for Hg^2+^ were achieved [22]. A modified carbon paste electrode based on multiwalled carbon nanotubes (MWCNTs) and 3-(4-methoxybenzylideneamino)-2-thioxothiazolodin-4-one as a new synthesized Schiff base was constructed for determination of Hg(II) and Pb(II) by square wave anodic stripping voltammetry and the detection limits were 0.18 *μ*g L^−1^ and 0.12 *μ*g L^−1^ for Hg(II) and Pb(II), respectively [23]. The sensor chemically grafted onto SiO_2_/Fe_3_O_4_ nanoparticles, as a modified core/shell structure, was successfully used for highly sensitive determination of trace amounts of Pb^2+^ with a detection limit of 0.21 *μ*g L^−1^ [24].

The present study used the differential pulse anodic stripping voltammetry (DPASV) technique combined with CMEs for the determination of Cu^2+^ and Pb^2+^ in aqueous solution.

## 2. Experiment

### 2.1. Reagents

All solutions were prepared with deionized water (Millipore, Milli Q) and all reagents used were of analytical grade.

The buffers used for the optimization study were as follows: acetate buffer, pH 4.47, and phosphate buffer, pH 6.82. The acetate buffer was prepared using a solution of HOAc 1.00 mol L^−1^ and a solution of NaOAc 1.00 mol L^−1^ in the proportion of 2 : 1 with ionic strength of 0.197 mol L^−1^. The phosphate buffer was prepared using a solution of Na_2_HPO_4_ 0.20 mol L^−1^ and a solution of NaH_2_PO_4_ 0.20 mol L^−1^ in the proportion of 3 : 2 with an ionic strength of 0.222 mol L^−1^. These buffers were used for pH control and as support electrolyte.

The sample was potable water from the Institute of Chemistry and Biotechnology of the University Federal of Alagoas, used without chemical treatment.

### 2.2. Apparatus (Instruments)

All the voltammetric measurements were performed using Autolab® Potentiostat/Galvanostat PGSTAT 12 (AUT73222) with an interface to a microcomputer system, which is controlled by GPES (General Purpose Electrochemical System) version 4.7, Eco Chemie B.V., Utrecht, Netherlands, and NOVA version 1.6, Metrohm Autolab B. V. 1.6.013, Copyright 2005–2012.

Electrochemical measurements were performed in a conventional electrochemical cell containing a three-electrode system such as glassy carbon (GC) electrode (BAS, diameter 3.0 mm) as a working electrode, Ag/AgCl (saturated KCl) as a reference electrode, and platinum wire as an auxiliary/counterelectrode.

The analysis of Scanning Electrochemical Microscopy was performed using a Scanning Electrochemical Microscope SECM.Net (23123), interfaced to a microcomputer system, which is controlled by SECM.Net (Scanning Electrochemical Microscope) version 2.2.58 Sensolytics GmbH, Bochum, Germany.

### 2.3. Synthesis of Nanomaterials

*Silica Gold Nanoparticles (AuNPs-SiO*_*2*_). The gold nanoparticles incorporated on mesoporous silica MCM-41 were synthesized by vigorously mixing 600 mL of deionized water with 6.0 mL NaOH solution (2.0 mol L^−1^), 1.34 mmol of hexadecyl trimethyl ammonium bromide (CTAB) in Teflon beaker (PTFE). After fifteen minutes, 12 mL of HAuCl_4_·3H_2_O solution (1%) and 3.0 mL of tetraethylorthosilicate (TEOS) (95%) were added. After addition of the TEOS, the temperature was kept at 50°C for 2 hours. The reaction product was isolated by filtration and washed with deionized water until being neutral (pH of the residual water equal to wash water). The solid obtained was dried at 80°C for 24 hours. The sample was calcined at 550°C for 4 h with a ramping rate of 5°C min^−1^ [25]. The gold nanoparticles in silica MCM-41 are about 13 nm in size after calcination at 550°C.

*Silica Nanoparticles (SiO*_*2*_ *NPs)*. The silica nanoparticles were synthesized in accordance with [26]. 50 mL of anhydrous ethanol, 3.6 mL of 28% ammonia, and 3.0 mL of TEOS were placed in a 100 mL round-bottomed flask and the solution was agitated at a constant speed of 300 rpm at 35°C. The silica nanoparticles were washed with water until a natural environment was achieved and then the pH was adjusted to 4.5–5.0 using HCl 0.01 M. The silica nanoparticle is approximately 100 nm in size.

### 2.4. Auxiliary Preparations

All solutions were prepared with deionized water but aqueous stock solutions of Pb^2+^ and Cu^2+^ ions were prepared by dilution in HCl 0.1 M of the respective standard 1000 mg L^−1^ solutions and solutions of HgSO_4_ in HCl 0.1 M, Bi(NO_3_)_3_ in HNO_3_ 1 M, and K_3_[Fe(CN)_6_]/K_4_[Fe(CN)_6_] 10^−3 ^M in 0.1 M KCl.

The multiwalled CNT and CNT/nanoAu-SiO_2_ nanomaterials were dispersed in DMF (N,N-dimethylformamide).

The materials and voltammetric cell were washed for 24 h with 10% nitric acid to minimize potential contamination.

### 2.5. Electrode Preparations

**Scheme 1:**
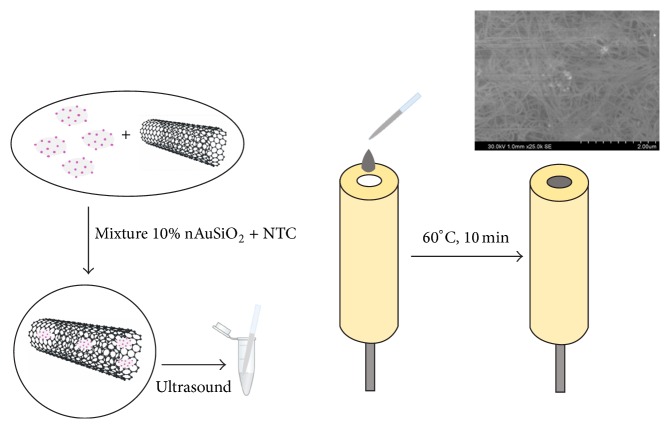
Schematic preparation of the modified electrode. Inset SEM image of CNT/nanoAu-SiO_2_.

The working electrode was prepared as follows: firstly, a glassy carbon electrode (*ϕ* = 3 mm) was polished carefully with 0.3 *μ*m alumina slurry, washed in deionized water and dried in air. Then a 1 *μ*L aliquot of nanomaterial solution was dropped onto the surface of the cleaned electrode and the electrode was kept at 70°C for 10 min. For multiwalled CNT, ten 1 *μ*L aliquots were dropped onto the surface of the electrode, for CNT/nanoAu-SiO_2_ ten aliquots (1 *μ*L) of CNT/nanoAu-SiO_2_ and for CNT/nanoSiO_2_ five aliquots (1 *μ*L) of CNT and five aliquots (1 *μ*L) of nanoSiO_2_ (Scheme 1).

### 2.6. Characterization of the Electrode Surface

Scanning Electrochemical Microscopy analysis of the GC/CNT, GC/CNT/Hg, GC/CNT/Bi, and GC/CNT/nanoAu-SiO_2_ electrode surface used to characterize processes and structural features at the substrate, as the tip is moved near the surface, was carried out using a two-electrode system, including a platinum ultramicroelectrode (*ϕ* = 10 *μ*m) as the working electrode and a platinum coil electrode (reference electrode + counterelectrode).

The electrochemical characteristics of the modified electrode surface were characterized using cyclic voltammetry (CV) with a standard solution of 5 mM (K_4_[Fe(CN)_6_]·3H_2_O) 0.1 M in KCl to check the electroactive area of the cleaned electrodes, sweeping the measurements from −0.2 to 0.6 V at 100 mV s^−1^ in 10 cycles (*versus* Ag/AgCl/Cl^−^ 3 mol/L). A scan array with sweep in comb mode was performed with *x* distance 1000 *μ*m, an increment of 10 *μ*m, and *y* distance 1000 *μ*m with 10 *μ*m team increment and 100 ms wait.

### 2.7. Electrochemical Measurements

The electrochemical characteristics of the modified electrode during the self-assembled process were characterized using cyclic voltammetry (CV) and differential pulse anodic stripping voltammetry (DPASV). Cyclic voltammetry used a standard K_3_[Fe(CN)_6_]/K_4_[Fe(CN)_6_] solution to examine the electroactive area of the cleaned electrodes, sweeping measurements from −0.3 to 0.6 V (*versus* Ag/AgCl/Cl^−^ 3 mol/L). The voltammograms of Pb^2+^ and Cu^2+^ ions were obtained by DPASV mode using two methods.

*Method 1*. It is as follows: first film formation and then determining the metal ion.

*Film Formation*. The electrochemical deposition of mercury and bismuth in the glassy carbon electrode was performed in an electrochemical cell with 10 mL of HgSO_4_ 10^−3 ^M and Bi (NO_3_)_3_ 10^−6 ^M swept from −1.0 V to −1.0 V for 300 s and 180 s, respectively, stirring and bubbling with argon gas. After this preconcentration step, the system was aborted.

*Metal Determination*. The DPV was performed in an electrochemical cell with 10 mL of HCl 0.1 M, aliquots of Cu^2+^, and Pb^2+^ concentration from 1 to 5 ppb with electrodeposition at 0.8 V (*versus* Ag/AgCl/Cl^−^ 3 mol/L) of 300 s and stirring and bubbling with argon gas. After this preconcentration step, the electrode was swept from −0.7 V to −0.3 V for Pb^+2^ and −0.45 V to −0.15 V for Cu^2+^ (anode region) with pulse amplitude 50 mV, pulse width 50 ms, and potential jump of 4 mV.

*Method 2*. It is as follows: film formation and determination of the metal performed concurrently. The DPV measurements were taken in an electrochemical cell with 10 mL of HCl 0.1 M containing 3.4 × 10^−6 ^M Hg^2+^ or Bi^3+^ 10^−6 ^M, with aliquots of 1 to 5 ppb concentration Cu^2+^ and Pb^2+^ and electrode positioned at −0.8 V (*versus* Ag/AgCl/Cl^−^ 3 mol/L) of 300 s for Hg^2+^, and Bi^3+^, and stirring and bubbling with argon gas. After this preconcentration step, the electrode was swept from 0.7 V to −0.3 V for Pb^2+^ and −0.45 V to −0.15 V for Cu^2+^ (anode region) with pulse amplitude 50 mV, pulse width 50 ms, and potential jump of 4 mV.

### 2.8. Interference Study

The influence of other species on the copper and lead ions in the voltammetric response of the electrode with gold nanoparticles was investigated by evaluating of the iron, zinc, and copper at a concentration 10 times greater compared to the analyte for calcium, 100 times greater compared to sodium, and 1000 times greater compared to sulfate and carbonate.

## 3. Results and Discussion

### 3.1. Morphological Characterization of Electrode Surface

There is great interest in obtaining information on the surface of the electrodes under study, since the electrochemistry is based on surface processes. The application of SECM is important for obtaining images that contain information on the electrochemical activity of the material and, in this study, the chemical, electrochemical, kinetic, and topographical properties of the interfaces.

**Figure 1:**
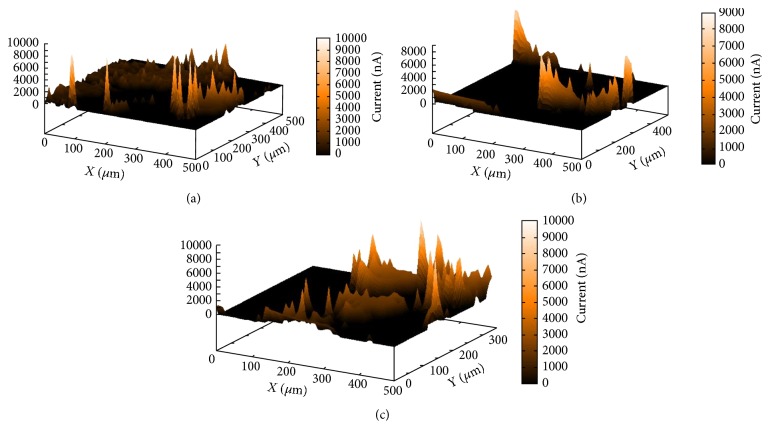
Analysis of electrochemical sweep microscopy of electrode surface (a) GC/CNT; (b) GC/CNT/Bi; and (c) GC/CNT/Hg.

Morphological analysis of the nanomaterial modified electrode surface has shown that these substrates have electroactivity that extends broadly across the surface of the electrodes, as can be seen in Figure 1(a). When electrodeposition of Hg and Bi metal is carried out, this electroactivity has a high current but does not extend over the whole electrode surface in the case of modification with CNT. See Figures 1(b) and 1(c).

This explains the electroactive performance of the nanomaterials and the films deposited on the glassy carbon. Although the whole surface of the electrode is not electroactive with the films, the high current produces good results.

### 3.2. Electrochemical Studies

**Figure 2:**
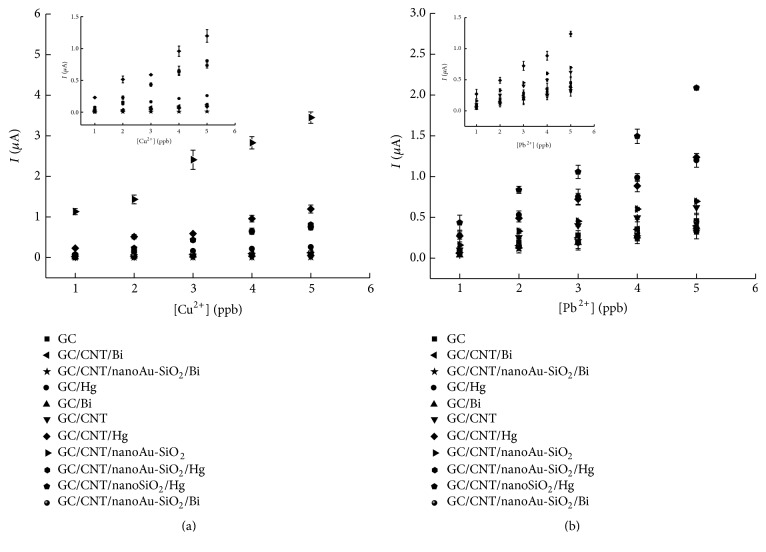
Graph comparing analytical calibration curves for analysis of Pb^2+^ and Cu^2+^ using glassy carbon electrode and its respective mobilizations with nanomaterials and metal films using Method 1. Simultaneous determination of ions Pb^2+^ and Cu^2+^ by DPASV GC/CNT/nanoAu-SiO_2_ (a) voltammogram and (b) analytical calibration curve for Pb^2+^, *r* = 0.9827, *I* = −2.2917 + 1.8224 [Pb^2+^], and, for Cu^2+^, *r* = 0.9571, *I* = −0.2395 + 0.2215 [Cu^2+^].

The electrochemical study of glassy carbon and nanomaterials, carbon nanotube (CNT), gold nanoparticles (nanoAu) and silica nanoparticles (nanoSiO_2_), and the metal films of mercury (Hg^2+^) and bismuth (Bi^3+^) to determine trace metal species, copper (Cu^2+^), and lead (Pb^2+^) was conducted using the differential pulse anodic stripping voltammetry technique (DPASV) following two methods: Method 1 and Method 2* (in line)*. Method 1 exhibits a redox signal, *Ip*_*a*_, directly proportional to the concentration of metal for all the electrode surfaces, as can be seen from the analytical calibration curves in Figure 2.

The data from the main figures, shown in Table 1, for each electrode, indicates that modification of the glassy carbon electrode with CNT/nanoAu-SiO_2_ provides good results in terms of correlation coefficient of linear correlation, sensitivity, linear band, and repeatability, compared with all the other modifications performed. The data reveals that CNT/nanoAu-SiO_2_ is highly sensitive, enabling detection of lower Cu^2+^ and Pb^2+^ concentrations compared to all other electrodes tested here. Compared with the recent literature, our electrode was better than Mo6SxI9-x NWs/GCE [27, 28] not only for copper but also for lead, with LOD and LOQ values for the CNT/nanoAu-SiO_2_ electrode lower than those for the Mo6SxI9-x NWs/GCE electrode.

**Figure 3:**
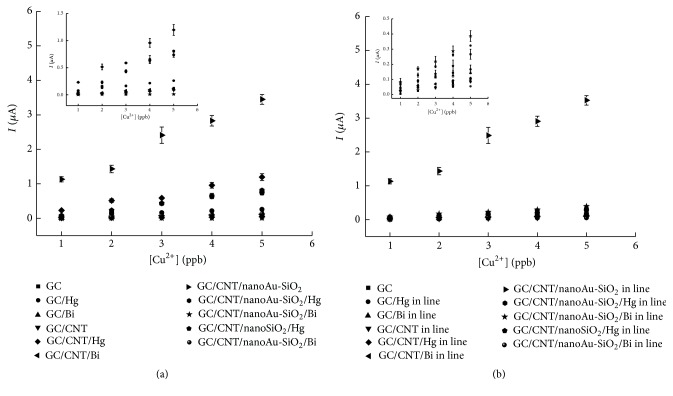
Graph comparing analytical calibration curves for analysis of Cu^2+^ using the glassy carbon electrode and its modifications with nanomaterials and metals. (a) Method 1. (b) Method 2.

The analytical calibration curves for the electrodes used for analysis of Cu^2+^ are plotted in Figure 3, for (a) Method 1 and (b) Method 2* (in line)*.

The electrodes in Method 1 showed greater sensitivity than that in Method 2. It is thus important to note that the concentration of metals used for formation of the film is different for the different methods, as the concentration of Hg^2+^ is 10^−3 ^mol/L in Method 1 and 3.4 × 10^−6 ^mol/L in Method 2 and, in the case of Bi^3+^, 10^−6 ^mol/L for both methods.

Figure 3(a) and Table 1 show that the CNT/nanoAu-SiO_2_ electrode exhibits a better analytical response using both methods, according to the analytical parameters examined. It is interesting to note the striking inclination of the line of this electrode (See Figure 3). One of the potential inorganic sorbent nanomaterials that could be used in water treatment is nanosilica. In addition to its high mechanical, thermal, and chemical resistance, it also is distinguished by its local availability and has many microsized pores on the surface to induce and adsorb various molecules or metals into the pore. Carbon nanotubes and nanogold have also shown interaction with various nanometals. In this study, the synergistic effect or the mixture of the three nanomaterials (CNT + Au-SiO_2_) promoted an increase of the sensitivity (modified electrode).

Studies have been conducted on the use of a chemically modified electrode (CME) and the performance of ASV is strongly affected by the electrode materials.  Table 2 shows this specifically for DPASV.

### 3.3. Simultaneous Determination of Cu^2+^ and Pb^2+^ Metal Ions

According to the electrochemical study to identify the best nanomaterial for determination of Cu^2+^ and Pb^2+^ metal ions, the GC/NTC/nanoAu-SiO_2_ electrode performed well and also exhibited good electroactivity, according to morphological analysis of the electrode surface using Scanning Electrochemical Microscopy (SECM), in such a way that this electrode was used for simultaneous determination of the species under study.

**Figure 4:**
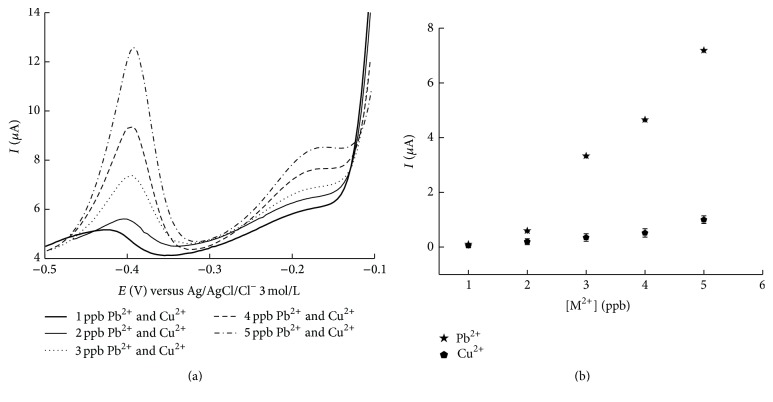
Simultaneous determination of Pb^2+^ and Cu^2+^ions by DPASV on GC/CNT/nanoAu-SiO_2_. (a) Voltammogram. (b) Analytical calibration curve, for Pb^2+^, *r* = 0.9827, *I* = − 2.2917 + 1.8224 [Pb^2+^], and for Cu^2+^, *r* = 0.9571, *I* = − 0.2395 + 0.2215 [Cu^2+^].

According to Figure 4(a), the voltammogram for simultaneous determination of Cu^2+^ and Pb^2+^, like the individual determination, showed that the redox signal, *Ip*_*a*_, is directly proportional to the concentration of the metals. The signal profile is the same, being broader for Cu^2+^ and narrower for Pb^2+^.

### 3.4. Study of Interferences

Interferences are substances that positively or negatively affect the size of the final measurement of quantitative analytical chemical method. The study of interferences was carried out under the same conditions of simultaneous analysis, with the following species used as possible interferences: Cd^2+^, Zn^2+^, and Fe^3+^ at concentrations ten times greater than the concentration of analytes, Na^+^ and Ca^2+^ at concentrations one hundred times greater than the concentration of analytes, and SO_4_^2−^ and CO_3_^2−^ at concentrations one hundred times greater than the concentration of analytes.

The presence of interferences ions in the electroanalytical system in determination of the lead and copper ions does not significantly interfere, since the stripping signal for the two metals is directly proportional to the concentration and retains the characteristic feature, although *Ep*_*a*_ for Pb^2+^ and Cu^2+^ subtly shifts to a more cathodic potential in simultaneous determination, with values of −0.450 V and −0.252 V in presence of Cd^2+^, −0.433 V and −0.236 V in presence of Zn^2+^, −0,450 V and −0,256 V in presence of Fe^3+^, −0,413 V and −0,207 V in presence of Na^+^  −0,421 V and −0,227 V in presence of Ca^2+^, −0,450 V and −0,248 V in presence of SO_4_^2−^, and −0,425 V and −0,240 V in presence of CO_3_^2−^.

Lead and copper ions may thus be characterized and quantified without interference, since the oxidation signal for both metals does not vary significantly with the addition of 10 ppb interferent Zn^2+^ ion to 1 ppb for Pb^2+^ and Cu^2+^.

### 3.5. Sample Analysis

The studies conducted to identify the best electrode and method were followed by optimization of the electroanalytical system and analysis of possible interferences for determination of Cu^2+^ and Pb^2+^. Water sample analysis used the GC/CNT/nanoAu-SiO_2_ electrode under optimized conditions, HCl 0.1 mol L^−1^, and a preconcentration time of 300 s, for determination of the Cu^2+^ and Pb^2+^ species.

The concentrations of analyte in the sample were determined by replacing the current values of the aliquots added (50 *μ*L, 75 *μ*L, 100 *μ*L, 125 *μ*L, and 150 *μ*L) in the analytical calibration curve of the Cu^2+^ and Pb^2+^ standard, determined under the same conditions as the sample analysis. The present study was unable to identify Pb^2+^ for reason of the absence of this metal ion in the sample.

**Figure 5:**
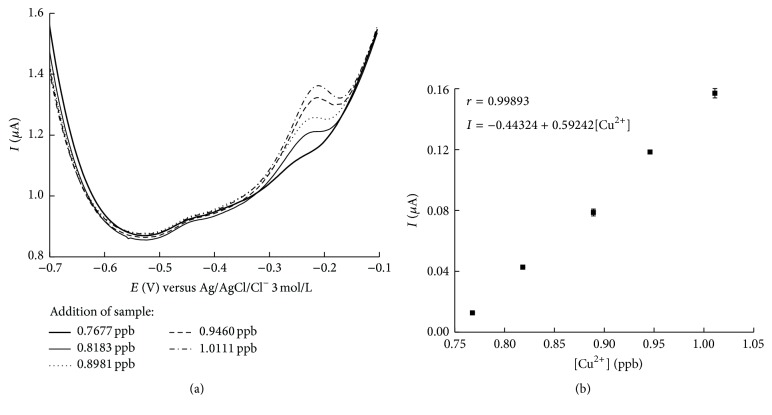
Determination of Cu^2+^ion by DPASV in GC/CNT/nanoAu-SiO_2_, in a sample of drinking water and in HCl pH 1 medium with preconcentration time of 300 s. (a) Voltammogram. (b) Analytical calibration curve.

The results presented in Figure 5 show that the electrode detects trace-level concentrations, in such a way that the sample has an oxidation signal with *Ip*_*a*_ directly proportional to the added concentration of analyte, with curve linearity of 0.989 and line equation *Ip*_*a*_ = − 0.44324 + 0.59242 [Cu^2+^]. A signal at *Ep*_*a*_ of approximately −0.211 V corresponds to the Cu^2+^ ion, with the broadened stripping signal feature characteristic of this metal.

According to ANVISA resolution 54/2000-ANVISA, for mineral and drinking water, the maximum permitted for copper is 1 mg/L. According to the results obtained in the study for copper in a sample of drinking water, this is in conformity with the established pattern.

### 3.6. Recovery Test

The recovery test was carried out by enriching the sample with a known quantity of the standard Cu^2+^ used – 10 ppb. The sample and the standard were put through all the same analytical procedures as the sample alone had been previously during the usual analytical procedure.

According to Table 3, recovery at the first three concentrations produced ideal results in an experimental band of error of 5% but this was not observed with the two subsequent concentrations, which varied from 10.70% to 18.43%, respectively. This shows that there is significant interference of the matrix in the sample in the electrochemical procedure using higher concentrations.

## 4. Conclusions

The present study investigated the electrochemical behavior of the nanomaterials carbon nanotube (CNT), nanogold (nanoAu), and nanosilica (nanoSiO_2_) and of metal films of mercury (Hg) and bismuth (Bi) in determination of trace metals lead (Pb) and copper (Cu) in an aqueous medium, using two methods, the common method and the in-line method.

The differential pulse anodic stripping voltammetry (DPASV) investigation showed the response of the current to be proportional to the concentration in the system, according to the characteristic electroactive area of each electrode substrate.

For the analytical parameters, linearity, sensitivity, repeatability, and linear band, nanoAu-SiO_2_ electrode performed the best in the analysis for determining Cu^2+^ and Pb^2+^ metals. This is very interesting, given the absence of a metal film.

Furthermore, the stripping of Pb^2+^ and Cu^2+^ on the nanoAu-SiO_2_ electrode established as adequate for the metals shows an anodic peak potential that facilitates oxidation compared to the other electrodes. With the nanoAu-SiO_2_ electrode, the oxidation of Pb^2+^ showed a stripping signal at *Ep*_*a*_ = −0.514 V and that of Cu^2+^ at *Ep*_*a*_ = −0.295 V.

Both of the methods used in this study may be applied, although the common method performs better for the determination of Pb and Cu metals with more sensitive results.

The morphological characterization of the surface of the carbon nanotube electrodes with or without the mercury and bismuth film and of the nanogold explains the satisfactory electrochemical results, given the high degree of electroactivity that these exhibit in accordance with their electronic properties.

The optimization of the electroanalytical system showed hydrochloric acid to be the best performing electrolyte and that the medium needs to be considerably acidic (pH 1) for arrangement of the species in free ions with a preconcentration time of 300 s to obtain a definite signal. The simultaneous determination of metals can successfully be based on the optimized system to obtain clearly separate and distinct oxidation signals.

The ions used in this study to determine the behavior of the lead and copper species generally do not interfere with the oxidation signal of metals, since they are obtained cleanly in their stripping potential and with the characteristic feature of the stripping signal of copper and lead.

## Figures and Tables

**Table 1:** Main figures for glassy carbon electrodes and their respective mobilizations with nanomaterials and metal films using Method 1 to analyze Cu^2+^ and Pb^2+^.

Electrode	Cu^2+^				Pb^2+^			
	LOD (ppb)	LOQ (ppb)	*r*	Equation	LOD (ppb)	LOQ (ppb)	*r*	Equation
GC	0.3896 ± 0.0220	1.2987 ± 0.0734	0.9974	*I* = −0.0189 + 0.0231 [Cu^2+^]	0.6608 ± 0.0177	2.2026 ± 0.0589	0.9992	*I* = 0.0063 + 0.0909 [Pb^2+^]
GC/Hg	0.5155 ± 0.0384	1.7182 ± 0.1279	0.9941	*I* = −0.0226 + 0.0582 [Cu^2+^]	0.3866 ± 0.0577	1.2887 ± 0.5179	0.9996	*I* = 0.0537 + 0.2328 [Pb^2+^]
GC/Bi	0.4243 ± 0.0334	1.4144 ± 0.1101	0.9993	*I* = −0.0089 + 0.0212 [Cu^2+^]	0.3597 ± 0.0571	1.1990 ± 0.1905	0.9949	*I* = −0.0460 + 0.0834 [Pb^2+^]
GC/NTC	0.3797 ± 0.0863	1.2658 ± 0.2878	0.9848	*I* = −8.4999 × 10^−5^ + 0.0158 [Cu^2+^]	0.4454 ± 0.0291	1.4848 ± 0.0269	0.9936	*I* = −0.0164 + 0.1347 [Pb^2+^]
GC/CNT/Hg	0.8246 ± 0.0538	2.7488 ± 0.1796	0.9932	*I* = 0.0492 + 0.1819 [Cu^2+^]	0.8714 ± 0.0589	2.9045 ± 0.1946	0.9952	*I* = 0.0069 + 0.2410 [Pb^2+^]
GC/CNT/Bi	0.3502 ± 0.1262	1.1673 ± 0.4209	0.9904	*I* = −0.0083 + 0.0257 [Cu^2+^]	0.3476 ± 0.0068	1.1587 ± 0.0238	0.9950	*I* = −0.0228 + 0.0863 [Pb^2+^]
GC/CNT/nanoAu-SiO_2_	0.3321 ± 0.0183	1.1073 ± 0.0610	0.9964	*I* = 0.4426 + 0.5930 [Cu^2+^]	0.2279 ± 0.0079	0.7595 ± 0.0263	0.9976	*I* = 0.0708 + 0.1258 [Pb^2+^]
GC/CNT/nanoAu-SiO_2_/Hg	0.6482 ± 0.0152	2.1606 ± 0.0507	0.9967	*I* = −0.2800 + 0.2777 [Cu^2+^]	1.0101 ± 0,0592	3.3670 ± 0,1974	0.9933	*I* = 0.0178 + 0.0594 [Pb^2+^]
GC/CNT/nanoAu-SiO_2_/Bi	2.7649 ± 0.1502	9.2166 ± 0.4545	0.9817	*I* = −2.1 × 10^−4^ + 0.0022 [Cu^2+^]	0.8772 ± 0,0580	2.9239 ± 0,0150	0.9956	*I* = −0.0123 + 0.0684 [Pb^2+^]
GC/CNT/nanoSiO_2_/Hg	0.3450 ± 0.0725	1.1501 ± 0.2664	0.9978	*I* = −0.0991 + 0.1739 [Cu^2+^]	0.4257 ± 0,0546	1.4191 ± 0.1820	0.9965	*I* = −0.0346 + 0.4228 [Pb^2+^]
GC/CNT/nanoSiO_2_/Bi	0.3629 ± 0.0042	1.2097 ± 0.0140	0.9831	*I* = −0.0146 + 0.0248 [Cu^2+^]	0.3315 ± 0.0763	1.1049 ± 0.2544	0.9964	*I* = −0.0359 + 0.0905 [Pb^2+^]

**Table 2:** Comparison of parameters and LOD of the different modified electrodes for determination of metal ions, including Cu(II) and Pb(II), using DPASV method.

Species	Electrolyte	Study electrode versus reference	Potential and deposition time	LOD (*μ*g L^−1^)	Reference
Pb^2+^, Cu^2+^, Cd^2+^, Tl^+^	0.1 M HCl	GC with HgF 25 mg L^−1^ and BiF 0,5 mg L^−1^ *versus* Ag/AgCl	−1.4 V for 60 s	HgFE 0.10, 0.15, 0.050, 0.70; BiFE 0.060,…, 0.043, 5.10 were found for Pb^2+^, Cu^2+^, Cd^2+^, Tl^+^, respectively.	De Carvalho et al., 2008 [29]
Pb^2+^, Cu^2+^, Cd^2+^, Hg^2+^	KCl pH 3	Modified carbon paste electrode based on BTPSBA^(b)^ *versus* saturated calomel electrode	−1.1 V for 300 s	8.28, 12.7, 50.4, 80.4 were found for Pb^2+^, Cu^2+^, Cd^2+^, Hg^2+^, respectively.	Cesarino et al., 2008 [30]
Pb^2+^, Cu^2+^, As^3+^, Hg^2+^	0.1 M HCl with 0.5 M NaCl	Au *versus* Ag/AgCl	−1.2 V for 32 s	0.2, 0.07, 0.4, 0.07 were found for Pb^2+^, As^3+^, Cu^2+^, Hg^2+^, respectively.	Alves et al., 2011 [31]
Zn^2+^, Cu^2+^, Hg^2+^, Pb^2+^	0.5 M NaCl with 1.0 mM HCl	Gold microwire *versus* Ag/AgCl	−2 V for 30 s	0.2, 0.3, 0.4, 0.4 for Zn^2+^, Cu^2+^, Hg^2+^, Pb^2+^, respectively.	Alves et al., 2013 [32]
Cu^2+^, Pb^2+^, Cd^2+^, Zn^2+^	0.5 M acetate buffer pH 1	BDD^(a)^ versus Ag/AgCl	−0.95 V for 240 s	0.37, 0.40, 1.28, 0.16 were found for Cu^2+^, Pb^2+^, Cd^2+^, Zn^2+^, respectively.	Honório et al., 2014 [33]
Pb^2+^, Cu^2+^, Cd^2+^	pH 4.5	CB-18-crown-6-GEC^(c)^ versus Ag/AgCl	−1.4 V for 120 s	1.5, 1.5, 2.4 were found for Pb^2+^, Cu^2+^, Cd^2+^, respectively.	Serrano et al., 2015 [27]
Cd^2+^, Pb^2+^, Cu^2+^	0.1 M pH 4.7 acetate buffer solution	Mo6SxI9-x NWs/GC^(d)^ versus saturated calomel electrode	−1.1 V for 240 s	0.10, 0.45, 0.20 were found for Cd^2+^, Pb^2+^, Cu^2+^, respectively.	Lin et al., 2015 [28]
Pb^2+^ and Cu^2+^	0.01 M KNO_3_ pH 4,5	SbSPCE^(e)^ versus Ag/AgCl	−0.7 V for 120 s	4,8 and 0,28 were found for Pb^2+^ and Cu^2+^, respectively.	Sosa et al., 2015 [34]
Cu^2+^, Hg^2+^, Pb^2+^	0.1 M NaCl pH 7	Polyviologen films versus Ag/AgCl	−0.7 V	63.0, 200.0, 207.0 were found for Cu^2+^, Hg^2+^, Pb^2+^, respectively.	Gadgil et al., 2016 [24]
Pb^2+^ and Cu^2+^	0.1 M HCl	GC/CNT/nanoAu-SiO_2_ *versus* Ag/AgCl	−0.8 V for 300 s	0.47 and 0.34 were found for Pb^2+^ and Cu^2+^, respectively.	This work

^(a)^BDD: boron-doped diamond.

^(b)^BTPSBA: 2-benzothiazolethiol organofunctionalized SBA-15 nanostructured silica.

^(c)^CB-18-crown-6-GEC: 4-carboxybenzo-18-crown-6 assisted by lysine on aryl diazonium salt monolayers anchored to the surface of graphite, epoxy composite electrode.

^(d)^Mo6SxI9-x NWs: molybdenum-chalcogenide-halide nanowires (NWs), which are composed of molybdenum (Mo), sulfur (S), and iodine (I) in the form of Mo6S9_xIx (MoSI).

^(e)^SbSPCE: antimony film screen-printed carbon electrode.

**Table 3:** Recovery test for Cu^2+^ in sample of drinking water.

Standard Cu^2+^	Water sample added	Recovered
10 ppb	0.7674 ppb	99.96%
	0.8261 ppb	100.95%
	0.9043 ppb	100.69%
	1.0473 ppb	110.70%
	1.1975 ppb	118.43%
